# Supplementary material for: Relationship of sleep and activity, assessed via a wristwatch-type pulsimeter with an accelerometer, with health status in community-dwelling older adults: A preliminary study
Source: PLoS One. 2025 Mar 5;20(3):e0317524. doi: 10.1371/journal.pone.0317524 (PMC11882082; doi:10.1371/journal.pone.0317524)
Supplement: Attachment — Submitted filename: PONE-D-24-27156_reviewer_reviewer comments.pdf [file pone.0317524.s001.pdf]

PLOS ONE

Relationship of sleep and activity, assessed via a wrist-watch-type pulsimeter with an accelerometer, with health status in community-dwelling older adults: A preliminary study

--Manuscript Draft--

**Manuscript Number:**	PONE-D-24-27156
**Article Type:**	Research Article
**Full Title:**	Relationship of sleep and activity, assessed via a wrist-watch-type pulsimeter with an accelerometer, with health status in community-dwelling older adults: A preliminary study
**Short Title:**	Sleep and activity via a wearable device in community-dwelling older adults
**Corresponding Author:**	Akiyo Sasaki-Otomaru<br>The Dia Foundation for Research on Ageing Societies<br>Tokyo, JAPAN
**Keywords:**	community-dwelling older adults; Frailty; Physical Activity; Sleep duration; wearable device
**Abstract:**	Wearable devices have the potential to promote a healthy lifestyle; however, studies on the use of wearable devices in monitoring health in older adults are limited. We aimed to investigate the relationship of sleep and activity data with health status among older adults. Fifty-five community-dwelling older adults were asked to wear a wristwatch-type wearable device (the Pulsense [PS]) and measure home blood pressure (HBP) over a period of 5–7 consecutive days. Deep sleep duration, physical and mental activity duration, and body movement duration were obtained from PS data using special software. We also collected data on demographics and physical and mental health status. We found that the body-movement duration in females was longer than that in males. The body-movement duration in men was negatively correlated with the Kihon Checklist (KCL) score and GDS and positively correlated with physical functioning, bodily pain, vitality, social function, and role emotional from the Medical Outcomes Survey Short Form-8 questionnaire, and hand-grip strength in a simple linear regression. There was no significant correlation between monitoring data and health status in women. In the multiple linear regression analysis, body-movement duration was negatively associated with age and the KCL score. KCL is a common questionnaire for screening frailty in Japan. Our results showed that body-movement duration was negatively associated with age and the KCL score, suggesting the potential of PS in guiding personalized health management of older community-dwelling adults with risks of frailty.
**Order of Authors:**	Akiyo Sasaki-Otomaru
	Kyoko Saito
	Kotaro Yamasue
	Osamu Tochikubo
	Yuka Kanoya
	Masahiko Inamori
**Additional Information:**	
**Question**	**Response**
**Financial Disclosure**<br><br>Enter a financial disclosure statement that describes the sources of funding for the work included in this submission. Review the submission guidelines for detailed	Yes

Powered by Editorial Manager® and ProduXion Manager® from Aries Systems Corporation

requirements. View published research articles from *PLOS ONE* for specific examples.<br><br>This statement is required for submission and **will appear in the published article** if the submission is accepted. Please make sure it is accurate.<br><br>**Funded studies**<br>Enter a statement with the following details:<br>• Initials of the authors who received each award<br>• Grant numbers awarded to each author<br>• The full name of each funder<br>• URL of each funder website<br>• Did the sponsors or funders play any role in the study design, data collection and analysis, decision to publish, or preparation of the manuscript?<br><br>Did you receive funding for this work?	
Please add funding details.<br>as follow-up to "**Financial Disclosure**<br><br>Enter a financial disclosure statement that describes the sources of funding for the work included in this submission. Review the submission guidelines for detailed requirements. View published research articles from *PLOS ONE* for specific examples.<br><br>This statement is required for submission and **will appear in the published article** if the submission is accepted. Please make sure it is accurate.	This work was supported by the 31st Foundation for Total Health Promotion and the Grant-in-Aid for Young Scientists (B), under Grant number. 17K17508 (KAKENHI).

Powered by Editorial Manager® and ProduXion Manager® from Aries Systems Corporation

**Funded studies**<br>Enter a statement with the following details:<br>• Initials of the authors who received each award<br>• Grant numbers awarded to each author<br>• The full name of each funder<br>• URL of each funder website<br>• Did the sponsors or funders play any role in the study design, data collection and analysis, decision to publish, or preparation of the manuscript?<br><br>Did you receive funding for this work?"	
Please select the country of your main research funder (please select carefully as in some cases this is used in fee calculation).<br>as follow-up to "**Financial Disclosure**<br><br>Enter a financial disclosure statement that describes the sources of funding for the work included in this submission. Review the submission guidelines for detailed requirements. View published research articles from *PLOS ONE* for specific examples.<br><br>This statement is required for submission and **will appear in the published article** if the submission is accepted. Please make sure it is accurate.<br><br>**Funded studies**<br>Enter a statement with the following details:<br>• Initials of the authors who received each award<br>• Grant numbers awarded to each author<br>• The full name of each funder<br>• URL of each funder website<br>• Did the sponsors or funders play any role in the study design, data collection and analysis, decision to publish, or preparation of the manuscript?<br><br>Did you receive funding for this work?"	JAPAN - JP
**Competing Interests**	The authors have declared that no competing interests exist.

Use the instructions below to enter a competing interest statement for this submission. On behalf of all authors, disclose any competing interests that could be perceived to bias this work—acknowledging all financial support and any other relevant financial or non-financial competing interests.<br><br>This statement is required for submission and **will appear in the published article** if the submission is accepted. Please make sure it is accurate and that any funding sources listed in your Funding Information later in the submission form are also declared in your Financial Disclosure statement.<br><br>View published research articles from *PLOS ONE* for specific examples.<br><br>**NO authors have competing interests**<br><br>Enter: *The authors have declared that no competing interests exist.*<br><br>**Authors with competing interests**<br><br>Enter competing interest details beginning with this statement:<br><br>*I have read the journal's policy and the authors of this manuscript have the following competing interests: [insert competing interests here]*<br><br>* typeset	
**Ethics Statement**<br><br>Enter an ethics statement for this submission. This statement is required if the study involved:<br><br>• Human participants<br>• Human specimens or tissue<br>• Vertebrate animals or cephalopods<br>• Vertebrate embryos or tissues	The research was conducted according to the World Medical Association Declaration of Helsinki. All participants provided written informed consent, and this cross-sectional study was approved by the Ethical Committee of the Yokohama City University School of Medicine (approval no: A200200005).

Powered by Editorial Manager® and ProduXion Manager® from Aries Systems Corporation

• Field research<br><br>Write "N/A" if the submission does not require an ethics statement.<br><br>General guidance is provided below. Consult the submission guidelines for detailed instructions. **Make sure that all information entered here is included in the Methods section of the manuscript.**	

Powered by Editorial Manager® and ProduXion Manager® from Aries Systems Corporation

**Format for specific study types**<br><br>**Human Subject Research (involving human participants and/or tissue)**<br>• Give the name of the institutional review board or ethics committee that approved the study<br>• Include the approval number and/or a statement indicating approval of this research<br>• Indicate the form of consent obtained (written/oral) or the reason that consent was not obtained (e.g. the data were analyzed anonymously)<br><br>**Animal Research (involving vertebrate animals, embryos or tissues)**<br>• Provide the name of the Institutional Animal Care and Use Committee (IACUC) or other relevant ethics board that reviewed the study protocol, and indicate whether they approved this research or granted a formal waiver of ethical approval<br>• Include an approval number if one was obtained<br>• If the study involved *non-human primates*, add *additional details* about animal welfare and steps taken to ameliorate suffering<br>• If anesthesia, euthanasia, or any kind of animal sacrifice is part of the study, include briefly which substances and/or methods were applied<br><br>**Field Research**<br><br>Include the following details if this study involves the collection of plant, animal, or other materials from a natural setting:<br>• Field permit number<br>• Name of the institution or relevant body that granted permission	
**Data Availability**<br><br>Authors are required to make all data underlying the findings described fully available, without restriction, and from the time of publication. PLOS allows rare exceptions to address legal and ethical concerns. See the PLOS Data Policy and FAQ for detailed information.	No - some restrictions will apply

A Data Availability Statement describing where the data can be found is required at submission. Your answers to this question constitute the Data Availability Statement and **will be published in the article**, if accepted.<br><br>**Important:** Stating 'data available on request from the author' is not sufficient. If your data are only available upon request, select 'No' for the first question and explain your exceptional situation in the text box.<br><br>Do the authors confirm that all data underlying the findings described in their manuscript are fully available without restriction?	
**Describe where the data may be found in full sentences. If you are copying our sample text, replace any instances of XXX with the appropriate details.**<br><br>• If the data are **held or will be held in a public repository**, include URLs, accession numbers or DOIs. If this information will only be available after acceptance, indicate this by ticking the box below. For example: *All XXX files are available from the XXX database (accession number(s) XXX, XXX.).*<br>• If the data are all contained **within the manuscript and/or Supporting Information files**, enter the following: *All relevant data are within the manuscript and its Supporting Information files.*<br>• If neither of these applies but you are able to provide **details of access elsewhere**, with or without limitations, please do so. For example:<br><br>*Data cannot be shared publicly because of [XXX]. Data are available from the XXX Institutional Data Access / Ethics Committee (contact via XXX) for researchers who meet the criteria for access to confidential data.*<br><br>*The data underlying the results presented in the study are available from (include the name of the third party*	The dataset generated and/or analyzed during the current study are not publicly due to ethical considerations. The data that support the findings of this study are available from the corresponding author upon reasonable request.

and contact information or URL).<br>• This text is appropriate if the data are owned by a third party and authors do not have permission to share the data.<br><br>* typeset	
Additional data availability information:	

Manuscript

Click here to access/download;Manuscript;manuscriptbodyPLOSOne0719.d

Title: Relationship of sleep and activity, assessed via a wrist-watch-type pulsimeter with an accelerometer, with health status in community-dwelling older adults: A preliminary study

Short title: Sleep and activity via a wearable device in community-dwelling older adults

Akiyo Sasaki-Otomaru [1,2]*, Kyoko Saito [3], Kotaro Yamasue [4], Osamu Tochikubo [4], Yuka Kanoya [2], and Masahiko Inamori [5]

[1] The Dia Foundation for Research on Ageing Societies, Tokyo, Japan

[2] Department of Gerontological Nursing, Yokohama City University School of Medicine Nursing Course, Yokohama, Japan

[3] Shukutoku University, Iruma, Japan

[4] Department of Public Health, Yokohama City University School of Medicine, Yokohama, Japan

[5] Department of Medical Education, Yokohama City University School of Medicine, Yokohama, Japan.

*Corresponding author

E-mail: sasaki@dia.or.jp

Abstract

Wearable devices have the potential to promote a healthy lifestyle; however, studies on the use of wearable devices in monitoring health in older adults are limited. We aimed to investigate the relationship of sleep and activity data with health status among older adults. Fifty-five community-dwelling older adults were asked to wear a wristwatch-type wearable device (the Pulsense [PS]) and measure home blood pressure (HBP) over a period of 5–7 consecutive days. Deep sleep duration, physical and mental activity duration, and body movement duration were obtained from PS data using special software. We also collected data on demographics and physical and mental health status. We found that the body-movement duration in females was longer than that in males. The body-movement duration in men was negatively correlated with the Kihon Checklist (KCL) score and GDS and positively correlated with physical functioning, bodily pain, vitality, social function, and role emotional from the Medical Outcomes Survey Short Form-8 questionnaire, and hand-grip strength in a simple linear regression. There was no significant correlation between monitoring data and health status in women. In the multiple linear regression analysis, body-movement duration was negatively associated with age and the KCL score. KCL is a common questionnaire for screening frailty in Japan. Our results showed that body-movement duration was negatively associated with

age and the KCL score, suggesting the potential of PS in guiding personalized health management of older community-dwelling adults with risks of frailty.

Introduction

Wearable devices have become a topic of interest in recent years [1]. These devices can measure vital signs such as heart rate, respiration rate, and body temperature, in addition to physical activity and sleep conditions. Accelerometers, which were once mainly worn on the waist, are increasingly being replaced by wrist-type models [2,3]. An accelerometer could evaluate not only moderate-to-vigorous physical activity but also low physical activity (LPA) and sedentary behavior; LPA accounted for a large proportion of total physical activity time among older adults with declining functionality [4]. The Canadian 24-Hour Movement Guideline provides evidence-based recommendations for a healthy day (24-h), comprising a combination of sleep, sedentary behaviors, and light-intensity and moderate-to-vigorous-intensity physical activity [5]. The wristwatch-type wearable devices are expected to provide 24-h monitoring.

We previously used a wristwatch-type pulsimeter with accelerometer (PS-500B; Pulsense®, Seiko Epson Co. Ltd., Nagano, Japan), which is an improvement and productization of previously reported models [6-8]. Our study showed that home blood

pressure (HBP) was affected by sleep, even after adjusting for age and body mass index (BMI) among healthy adults [8]. Therefore, we considered that wearable devices can be used to comprehensively assess the lifestyle of older adults.

Japan has the world's highest life expectancy and a persistently low birth rate. Japan's population is aging more rapidly than that of any other country [9]. With the increasing number of older adults, a substantial increase in health care and social security costs is expected. The ratio of individuals in need of nursing care increases because of dementia and physical functional deterioration. In Japan, the main reasons for needing nursing care are dementia, cerebrovascular disease, and frailty [10]; therefore, healthy behaviors, such as increased physical activity and improved quality of sleep, are important factors to prevent these diseases and symptoms.

The use of wristwatch-type devices has the potential to promote healthy lifestyles in terms of physical activity and weight [11,12]. However, studies on the use of wearable devices in monitoring health in older adults are limited [13,14]. This study aimed to examine the relationship of sleep and activity data (obtained using Pulsense) with the health status of community-dwelling older adults.

Materials and methods

Participants

From July 2015 to August 2017 and from June 2020 to October 2020, we recruited community-dwelling older adults participating in health classes at local health centers. The eligibility criteria were as described in the previous study [8]. Of the 61 participants in the present study, six were excluded due to a lack of sleep and activity data. Finally, we included 55 older adults.

Ethical considerations

The research was conducted according to the principles of the World Medical Association Declaration of Helsinki. Informed consent was obtained from all participants, and this cross-sectional study was approved by the Ethical Committee of the Yokohama City University School of Medicine (approval no: A150326011 & A200200005).

Variables

Sleep and activity

Sleep and activity data were measured using the Pulsense (henceforth termed PS) and processed using special software to yield data on deep-sleep, physical-activity, mental-activity, and body-movement durations [8]. The participants wore the PS over a period of 5–7 consecutive days and nights, as described in a previous study [8].

HBP

HBP was measured twice a day, in the morning and at night, in a sitting position after at least 10 min of rest according to the guidelines, and the data were averaged over a 5- to 7-day period for each participant [15]. Hypertension was defined as the use of antihypertensive drugs, systolic blood pressure (SBP) ≥135 mmHg, and/or diastolic blood pressure (DBP) ≥85 mmHg.

Kihon Checklist and depression

The Kihon Checklist (KCL) score was used for a comprehensive evaluation of the physical, psychological, functional, and social status of the participants. The KCL consists of five subdomains (physical, nutritional, oral, cognitive function, and depressive mood). Each KCL score indicates the perceived level of difficulty of the activity in the question, and a higher score on the checklist indicates a higher risk of requiring support in each domain. A total score of ≥6 indicated frailty [16]. The current study applied the KCL without the depression score [17]. Instead, the Geriatric Depression Test-5 (GDS-5) was used to evaluate depressive mood, according to a previous study [16]. A higher score on the GDS indicated a higher risk of depression [18].

Subjective sleep

Subjective sleep was assessed using the Pittsburgh Sleep Quality Index (PSQI), which included 19 self-administered questions that covered seven domains, namely subjective

quality, sleep latency, sleep duration, habitual sleep efficiency, sleep disturbance, sleep medication use, and daytime dysfunction. Sleep disturbance was defined as having a PSQI score ≥6 [19].

Health-related quality of life

Health-related quality of life was assessed by administering the Medical Outcomes Survey Short Form-8 questionnaire (SF-8) [20]. The SF-8 consisted of eight items, each representing one health profile dimension: general health perception, physical functioning, role functioning–physical, bodily pain, vitality, social functioning, mental health, and role functioning–emotional. The Japanese version of the SF-8 met the standard criteria for content, construct, and criterion validity, as it was based on a nationwide survey [20].

Physical function

Measures of physical function included gait speed (in m/s) and hand-grip strength (in kg) and were assessed to reflect physical functional status. To evaluate gait speed, the participants were asked to walk 11 m at their usual pace, and the time taken to traverse the middle 5 m of this distance was measured once [21]. The recorded times were converted to speed (in m/s). Hand-grip strength (kg) was measured using a digital hand dynamometer (T.K.K.5401; Takei Scientific Instruments Co., Ltd., Niigata, Japan). The grip strength of each hand was assessed twice, and the maximal value of each hand was

averaged for analysis.

Statistical analysis

All data were analyzed using SPSS Statistics version 28 (IBM Corp., Chicago, IL, USA). The normality of the data distribution was tested using the Shapiro–Wilk test. The KCL score, PSQI score, GDS score, physical activity duration, and mental activity duration data were not normally distributed. Therefore, differences between the sexes were compared using the Mann–Whitney U-test. Spearman's correlation test was used to assess the relationship between the PS data and health status measurements, including HBP. Lastly, multiple linear regression analyses were used to explore the association between the PS data (deep-sleep and body-movement durations) and variables identified as significant by the chi-square test and Spearman's correlation test. All analyses were two-sided at the 5% level of significance.

Results

Participant characteristics

Of the 55 participants, 35 (63.6%) were female and 26 (47.3%) had hypertension. The mean age of the participants was 73.8 years. Table 1 compares the characteristics of the participants according to sex. There were significant differences in age, PSQI score, hand-grip strength, and body-movement duration between men and women.

Table 1. Characteristics of participants.

	Male					Female					
Parameters	n	mean	median	min	max	n	mean	median	min	max	P
Age (years)	20	76.0	76.0	67.0	91.0	35	72.6	73.0	65.0	82.0	0.032*
BMI (kg/m2)	20	23.2	22.9	17.7	31.2	35	21.7	21.8	15.5	32.8	0.144
Living status (single; n, %)		1	(11.1)				8	(88.9)			0.085
Kihon Checklist	20	3.4	2.5	0.0	12.0	35	3.7	4.0	0.0	8.0	0.457
(Total score; No. 1–20)											
Kihon Checklist (6 or more)		3	(15.0)				8	(22.9)			0.483
GDS5	20	0.9	0.0	0.0	4.0	35	0.6	0.0	0.0	3.0	0.814
Hypertension (n, %)		11	(55.0)				15	(45.5)			0.279
Use of antihypertensive drugs		7	(35.0)				10	(28.6)			0.420
(n, %)											
SF-8 Physical functioning	20	47.89	50.27	34.38	63.38	35	49.79	50.27	26.89	63.38	0.366
Role physical	20	48.53	53.54	16.69	53.54	35	48.37	47.77	27.59	53.54	0.133
Bodily pain	20	50.46	54.09	21.80	54.09	35	50.09	54.09	27.91	54.09	0.378
General health	20	50.78	52.46	38.21	60.35	35	48.28	46.10	38.21	60.35	0.174
Vitality	20	49.78	53.74	28.68	60.01	35	50.85	53.74	38.51	60.01	0.840

Social functioning	20	51.08	55.14	26.00	55.14	35	51.69	55.14	37.65	55.14	0.347
Role emotional	20	51.95	53.83	36.30	56.93	35	51.81	50.72	36.30	56.93	0.800
Mental health	20	51.24	54.19	31.42	54.19	35	51.41	54.19	42.24	54.19	0.540
PSQI	20	4.5	4	0	11	35	7.4	7	0	15	0.009*
PSQI scores more than 6 (n, %)		7	(35.0)				20	(57.1)			0.114
Hand-grip strength (kg)	20	33.6	34.9	18.0	45.8	35	21.9	21.3	15.5	32.3	<0.001*
Walking-speed (m/s)	19	1.5	1.4	0.6	2.5	35	1.5	1.5	1.0	2.0	0.458
PS Deep-sleep duration (h)	20	4.26	3.98	1.76	7.75	35	4.97	5.00	2.06	8.92	0.080
Physical-activity duration (h)	20	2.17	2.09	0.14	5.77	35	2.36	2.21	0.35	7.63	0.643
Mental-activity duration (h)	20	0.99	0.66	0.08	3.81	35	0.74	0.62	0.04	3.10	0.489
Body-movement duration (h)	20	6.01	5.57	3.42	8.89	35	7.09	7.15	4.73	9.81	0.026*

PSQI, Pittsburgh Sleep Quality Index; PS, a pulsimeter (Pulsense®); GDS-5, Geriatric Depression Test-5; SF-8, Medical Outcomes Survey Short Form-8 questionnaire

Mann–Whitney U test

* $p < 0.05$

PS data and health status

Spearman's rank correlation coefficients for correlations of sleep and activity data with other variables in each sex are shown in Table 2 and 3. In male participants, physical activity duration correlated negatively with KCL score and GDS5 and positively with physical functioning, role physical, bodily pain, vitality, social functioning in the SF-8, hand-grip strength, and walking speed. Body-movement duration correlated positively with physical functioning score, role physical, bodily pain, vitality, social functioning, and role emotional in the SF-8, hand-grip strength, and walking speed. However, body-movement duration correlated negatively with KCL score and GDS5. Deep sleep duration correlated positively with BMI in men.

On the other hand, there was no significant correlation between PS data and health status among female participants.

Table 2. Spearman's rank correlation coefficients between sleep and activity data and variables in male participants (n=20).

		Deep sleep duration	Physical activity duration	Mental activity duration	Body movement duration
Age	rs	-0.200	-0.187	-0.077	-0.280
	p	0.398	0.430	0.747	0.231
BMI	rs	0.522	-0.166	-0.275	-0.215
	p	0.018*	0.484	0.241	0.362
Kihon Checklist score	rs	-0.068	-0.793	-0.311	-0.701
	p	0.775	<.001*	0.182	<.001*
GDS5	rs	0.203	-0.463	-0.011	-0.538
	p	0.392	0.040*	0.963	0.014*
SF-8 Physical functioning	rs	0.004	0.652	0.126	0.779
	p	0.986	0.002*	0.598	<.001*
Role physical	rs	-0.182	0.511	0.236	0.469
	p	0.442	0.021*	0.315	0.037*
Bodily pain	rs	-0.182	0.511	0.236	0.469
	p	0.442	0.021*	0.315	0.037*
General health	rs	0.230	0.206	0.240	0.210

		p	0.329	0.384	0.307	0.375
	Vitality	rs	-0.083	0.635	0.239	0.610
		p	0.729	0.003*	0.310	0.004*
	Social functioning	rs	-0.223	0.568	0.189	0.522
		p	0.345	0.009*	0.425	0.018*
	Role emotional	rs	-0.069	0.348	-0.019	0.473
		p	0.772	0.133	0.938	0.035*
	Mental health	rs	-0.148	0.443	0.169	0.385
		p	0.532	0.050	0.476	0.094
PSQI		rs	0.179	-0.019	0.171	-0.114
		p	0.449	0.937	0.471	0.634
Hand-grip strength		rs	-0.263	0.462	-0.038	0.614
		p	0.262	0.040*	0.875	0.004*
Walking-speed		rs	-0.125	0.460	0.167	0.465
(n=19)		p	0.611	0.048*	0.495	0.045*

GDS-5, Geriatric Depression Test-5; PSQI, Pittsburgh Sleep Quality Index; SF-8, Medical Outcomes Survey Short Form-8 questionnaire, SBP, Systolic blood pressure; DBP, Diastolic blood pressure

*p<0.01

Table 3. Spearman's rank correlation coefficients between sleep and activity data and variables in female participants (n=35).

		Deep sleep duration	Physical activity duration	Mental activity duration	Body movement duration
Age	rs	0.284	-0.289	-0.257	-0.254
	p	0.098	0.093	0.135	0.141
BMI	rs	-0.040	-0.319	-0.317	-0.080
	p	0.820	0.062	0.063	0.650
Kihon Checklist score	rs	-0.062	-0.017	0.149	-0.284
	p	0.725	0.925	0.393	0.098
GDS5	rs	-0.003	-0.108	-0.015	0.028
	p	0.987	0.535	0.931	0.874
SF-8 Physical functioning	rs	0.222	-0.211	-0.207	<.001
	p	0.200	0.223	0.233	0.999
Role physical	rs	0.079	-0.127	-0.198	0.080

		p	0.653	0.466	0.253	0.649
	Bodily pain	rs	0.009	-0.072	-0.080	-0.094
		p	0.958	0.682	0.647	0.591
	General health	rs	0.151	-0.105	0.010	-0.127
		p	0.386	0.548	0.953	0.469
	Vitality	rs	0.133	-0.177	-0.188	0.012
		p	0.446	0.310	0.281	0.947
	Social functioning	rs	0.091	-0.079	-0.160	0.059
		p	0.603	0.653	0.358	0.737
	Role emotional	rs	0.056	-0.017	-0.122	0.090
		p	0.747	0.923	0.486	0.607
	Mental health	rs	-0.058	0.053	-0.081	0.101
		p	0.740	0.761	0.642	0.563
PSQI		rs	-0.029	0.142	0.279	-0.084
		p	0.868	0.417	0.104	0.632
Hand-grip strength		rs	0.129	-0.130	-0.106	-0.042
		p	0.461	0.457	0.545	0.812
Walking-speed		rs	0.114	0.101	-0.054	0.320

	p	0.516	0.565	0.760	0.061

GDS-5, Geriatric Depression Test-5; PSQI, Pittsburgh Sleep Quality Index; SF-8, Medical Outcomes Survey Short Form-8 questionnaire, SBP, Systolic blood pressure; DBP, Diastolic blood pressure

*p<0.01

Association between body-movement duration and health status

Multilinear regression analyses were conducted to explore the associations between demographic factors and body-movement duration. Table 4 shows that the KCL score emerged as the strongest independent predictor of body movement duration (beta = -0.437, p <0.001). No significant model could be established for factors influencing deep-sleep duration after multiple regression analysis for deep sleep duration and independent variables.

Table 4. Multilinear regression analysis between body movement duration and independent variables (n=55).

	β	95% CI		p
Age	-0.274	-0.15	-0.008	0.040*
BMI	-0.120	-0.154	0.055	0.347
KCL	-0.437	-0.409	-0.124	<.001*

PSQI scores	-0.026	-0.105	0.085	0.832
Hypertension	-0.113	-1.116	0.417	0.364
Sex (male =1)	-0.250	-1.642	0.036	0.060
Adusted-R^2	0.370			
P	<0.001			

BMI, body mass index; KCL, Kihon Checklist; PSQI, Pittsburgh Sleep Quality Index; CI, confidence interval

* $p < 0.05$

Discussion

The present study used physical and sleep data using PS. Our results showed that body movement duration decreased in correlation with the higher risk of frailty in older adults after adjusting for sex. Frailty prevention is an important topic, particularly concerning Japanese older adult women [22]. The KCL is a governmental standardized index assessing frailty in Japanese individuals [23].

The body-movement duration in women was longer than that in men. The body-movement duration assessed by the PS included light physical activity, such as housework and cooking [24]. A nationwide survey in Japan reported that older women spent more

time on housework compared to older men [25]. Another previous study showed that women spent more time in LPA compared to men [4]. Additionally, a cross-sectional study showed that light physical activity was positively associated with physical performance [26]. Light physical activity indicates 2.0–2.9 metabolic equivalents (METs) of physical activity, which includes cooking, feeding household animals, or doing laundry [27]. These activities might be easy to adopt in the elderly lifestyle by both sexes. Although body-movement duration by PS did not imply LPA, PS might be a useful evaluator of light physical activity to set a target for maintaining and/or increasing body-movement duration. This is so that older adults can understand their own activity time and adjust it as needed.

PS also evaluates physical and mental activities. In this study, physical and mental activity data were not normally distributed; therefore, we did not conduct a multiple linear regression analysis. In simple linear regression analysis, physical-activity duration correlated negatively with the KCL and GDS scores and positively with several items of the SF-8. Both physical- and mental-activity durations might be associated with health status; however, further research is needed to determine the specific factors that influence activity duration. Sedentary behavior in older adults has been linked with negative health outcomes [28]; older people are not always able to exercise because of

underlying health conditions. However, PS evaluates acceleration and pulse. Future research should explore mental-activity duration using PS to identify favorable sedentary behavior patterns.

Deep sleep duration correlated positively with BMI in simple linear regression in men. While short sleep duration is generally associated with an increased risk of obesity [29,30], a study reported that long sleep duration was associated with sarcopenic obesity in older men [31]. In our study, we did not find a significant model when deep sleep duration was used as the dependent variable in a multiple regression analysis. This suggested that further investigation was needed.

In addition, a gender-specific approach is necessary to maintain and improve activity duration. Men spend their time on various activities, such as work, housework, and hobbies. Further reducing these activity times can have negative effects on their physical and mental health. Therefore, it is important to avoid further reducing time to maintain activity levels. Women have more reasons for decreased activity levels than men, such as injuries from falls and fractures. It is important to examine these reasons individually and take appropriate measures. For older adults, it is particularly important for family and caregivers to cooperate in monitoring physical activity duration. Family members living far away can check on the older adult's condition by phone or video call

and encourage them to exercise as needed. Caregivers can also observe and record the older adult's activity time in their daily lives.

Our study had some limitations. First, only a small number of participants were included, and the participants were health-conscious. Second, although sleep and activity durations may be affected by the season of the year, the data were collected over periods throughout the year due to weather considerations. This resulted in a survey that was not a comprehensive daily 1-week assessment. Third, we did not collect information on the nutritional status, which could notably influence frailty. Lastly, the PS could measure only activity and sleep. Other wearable devices with oximeters, electrocardiograms, and other functions are also currently available and warrant further research.

Conclusion

This cross-sectional study suggests that a decrease in body movement duration may be a risk factor for frailty in older adults. Monitoring physical activity duration and body movement duration by PS could be important data not only for older adults themselves but also for their families, especially those living far away, and informal caregivers who cannot observe them constantly.

Acknowledgment

We would like to thank all participants and the staff of local health centers. We thank Ms. Kaori Yoshida from Gerontological Nursing at Yokohama City University for her assistance in data collection.

References

1. Piwek L, Ellis DA, Andrews S, Joinson A. The rise of consumer health wearables: promises and barriers. PLoS Med. 2016;13: e1001953. doi: 10.1371/journal.pmed.1001953.

2. Strain T, Wijndaele K, Dempsey PC, Sharp SJ, Pearce M, Jeon J, et al. Wearable device measured physical activity and future health risk. Nat Med. 2020;26: 1385–1391. doi: 10.1038/s41591-020-1012-3.

3. Doherty A, Jackson D, Hammeria N, Plötz T, Olivier P, Granat MH, et al. Large scale population assessment of physical activity using wrist worn accelerometers: The UK Biobank Study. PLoS One. 2017;12: e0169649. doi: 10.1371/journal.pone.0169649.

4. Amagasa S, Inoue S, Ukawa S, Sasaki S, Nakamura K, Yoshimura A, et al. Are Japanese women less physically active than men? Findings from the DOSANCO Health Study. J Epidemiol. 2021;31: 530–536. doi: 10.2188/jea.JE20200185.

5. Ross R, Chaput JP, Giangregorio LM, Janssen I, Saunders TJ, Kho ME, et al. Canadian 24-hour movement guidelines for adults aged 18-64 years and adults aged 65 years or older: an integration of physical activity, sedentary behaviour, and sleep. Appl Physiol Nutr Metab. 2020;45: S57–S102. doi: 10.1139/apnm-2020-0467.

6. Ono K, Yamasue K, Tochikubo O, Terauchi Y, Mizushima S. Lifestyle monitoring with the use of an earphone-type thermometer, and ambulatory blood pressure monitoring and a new wrist watch-type pulsimeter with accelerometer. Clin Exp Hypertens. 2014;36: 97–102. doi: 10.3109/10641963.2014.892120.

7. Sawai A, Fujii H, Sato Y, Takuma A, Utsumi M,Sawai S, et al. Development of wristwatch-type stress-caloriemonitor and its wireless communication management system. Gazz Med Ital. 2016;175: 442–445.

8. Sasaki-Otomaru A, Yamasue K, Tochicubo O, Saito K, Inamori M. Association of home blood pressure with sleep and physical and mental activity, assessed via a wristwatch-type pulsimeter with accelerometer in adults. Clin Expl Hypertens. 2020;42: 131–138. doi: 10.1080/10641963.2019.1590382.

9. Arai H, Ouchi Y, Toba K, Endo T, Shimokado K, Tsubota K, et al. Japan as the front-runner of super-aged societies: perspectives from medicine and medical care in Japan. Geriatri Gerontol Int. 2015;15: 673–687. doi: 10.1111/ggi.12450.

10. Annual Report on the Aging Society (in Japanese). Cabinet Office Japan, 2019. [Cited 30 Aug 2023]. Available from: https://www8.cao.go.jp/kourei/whitepaper/w-2019/html/zenbun/s1_2_2.html.

11. Ringeval M, Wanger G, Denford J, Pare G, Kitsiou S. Fitbit-based interventions for healthy lifestyle outcomes: systematic review and meta-analysis. I Med Internet Res. 2020;22: e23954. doi: 10.2196/23954: 10.2196/23954.

12. Jakicic JM, Davis KK, Rogers RJ, King WC, Marcus MD, Helsel D, et al. Effect of wearable technology combined with a lifestyle intervention on long-term weight loss: the IDEA Randomized Clinical Trial. J Am Med Assoc. 2016;316: 1161–1171. doi: 10.1001/jama.2016.12858.

13. Kekade S, Hseieh CH, Islam MM, Atique S, Mohammed Khalfan A, Li YC, et al. The usefulness and actual use of wearable devices among the elderly population. Comput Meth Prog Bio. 2018;153: 137–159. doi: 10.1016/j.cmpb.2017.10.008.

14. Boulton E, Hawley-Hague H, French DP, Mellone S, Zacchi A, Clemson L, et al. Implementing behavior change theory and techniques to increase physical activity and prevent functional decline among adults aged 61-70: the PreventIT project. Prog Cardiovasc Dis. 2019;62: 147–156. doi: 10.1016/j.pcad.2019.01.003.

15. Shimamoto K, Ando K, Fujita T, Hasebe N, Higaki J, Horiuchi M, et al. The Japanese

Society of Hypertension Guideline for the Management of Hypertension (JSH 2014). Hypertens Res. 2014;37: 253–390. doi: 10.1038/hr.2014.20.

16. Ogawa K, Fujiwara Y, Yoshida H, Nishi M, Fukaya T, Kim M, et al. [The validity of the“Kihon Check-list” as an index of frailty and its biomarkers and inflammatory markers in elderly people]. Nihon Ronen Igakkai Zasshi. 2011;48: 545–552. doi: 10.3143/geriatrics.48.545.

17. Kamegaya T, Yamaguchi H, Hayashi K. Evaluation by the Basic Checklist and the risk of 3 years incident long-term care insurance certification. J Gen Fam Med. 2017;18: 230–236. doi: 10.1002/jgf2.52.

18. Hoyl MT, Alessi CA, Harker JO, Josephson KR, Pietruszka FM, Koelfgen M, et al. Development and testing of a five-item version of the Geriatric Depression Scale. J Am Geriatr Soc. 2019;47: 873–878. doi: 10.1111/j.1532-5415.1999.tb03848.x.

19. Doi Y, Minowa M, Uchiyama M, Okawa M, Kim K, Shibui K, et al. Psychometric assessment of subjective sleep quality using the Japanese version of the Pittsburgh Sleep Quality Index (PSQI-J) in psychiatric disordered and control subjects. Psychiatry Res. 2000;97: 165–172. doi: 10.1016/s0165-1781(00)00232-8.

20. Fukuhara S, Suzukamo Y. Manual of the SF-8 Japanese edition. Kyoto: Institute for Health Outcomes & Process Evaluation Research, Advances in Applied Sociology. 2004.

21. Taniguchi T, Yoshida H, Fujiwara Y, Motohashi Y, Shinkai S. A prospective study of gait performance and subsequent cognitive decline in a general population of older Japanese. J Gerontol Series A Biol Sci. 2012;67: 796–803. doi: 10.1093/gerona/glr243.

22. Kojima G, Iliffe S, Taniguchi Y, Shimada H, Rakugi H, Walters K. Prevalence of frailty in Japan: a systematic review and meta-analysis. J Epidemiol. 2017; 27:347–353. doi: 10.1016/j.je.2016.09.008.

23. Satake S, Senda K, Hong YJ, Miura H, Endo H, Sakurai T, et al. Validity of the Kihon Checklist for as-sessing frailty status. Geriatr Gerontol Int. 2016;16: 709–715. doi: 10.1111/ggi.12543.

24. Yoshida K, Sasaki-Otomaru A, Kanoya Y, Saito K, Inamori M. Effect of feedback on the physical activity of community-dwelling, older adults (in Japanese). J Japan Health Med Assoc. 2021;30: 361–371. doi: 10.20685/kenkouigaku.30.3_361.

25. Portal Site of Official Statistics of Japan. 2016 Survey on time use and leisure activities. [Cited 30 Aug 2023]. Available from: https://www.e-stat.go.jp/en/stat-search?page=1&toukei=00200533 2016.

26. Izawa KP, Shibata A, Ishii K, Miyawaki R, Oka K. Associations of low-intensity light physical activity with physical performance in community-dwelling elderly Japanese: a cross-sectional study. PLoS One. 2017;12: e0178654. doi: 10.1371/journal.pone.0178654.

27. Ainsworth BE, Haskell WL, Whitt MC, Irwin ML, Swartz AM, Strath SJ, et al. Compedium of physical activities: an update of activity codes and MET intensities. Med Sci Sports Exerc. 2000;43: 1575–1581. doi: 10.1097/00005768-200009001-00009.

28. De Rezende LFM, Rey-Lopez JP, Matsudo VKR, do Carmo Luiz O. Sedentary behavior and health outcomes among older adults: a systematic review. BMC Public Health. 2014;14: 333. doi: 10.1186/1471-2458-14-333.

29. Chien MY, Wang Y, Chen HC. The Relationship of Sleep Duration with Obesity and Sarcopenia in Community-Dwelling Older Adults. Gerontology. 2015;61: 399-406. doi: 10.1159/000371847.

30. Katsuura-Kamano S, Arisawa K, Uemura H, Nguyen TV, Takezaki T, Ibusuki R, et al. Association of skipping breakfast and short sleep duration with the prevalence of metabolic syndrome in the general Japanese population: Baseline data from the Japan Multi-Institutional Collaborative cohort study. Prev Med Rep. 2021;24: 101613. doi: 10.1016/j.pmedr.2021.101613.

31.Yang M, Zhang Y, Zhao W, Ge ML, Sun XL, Jia SL, et al. Association of sleep duration with sarcopenic obesity in multi-ethic older adults: findings from the WCHAT study. BMC Geriatrics. 2022;22: 899. doi: 10.1186/s12877-022-03543-0.
