# Supplementary material for: Relationship of sleep and activity, assessed via a wristwatch-type pulsimeter with an accelerometer, with health status in community-dwelling older adults: A preliminary study
Source: PLoS One. 2025 Mar 5;20(3):e0317524. doi: 10.1371/journal.pone.0317524 (PMC11882082; doi:10.1371/journal.pone.0317524)
Supplement: Attachment — Submitted filename: renamed_0e7ab.docx [file pone.0317524.s002.docx]

Dear Dr. Hidetaka Hamasaki,

Thank you for the thoughtful and constructive feedback on our manuscript, “Relationship of sleep and activity, assessed via a wristwatch-type pulsimeter with an accelerometer, with health status in community-dwelling older adults: A preliminary study.” We have addressed the reviewer’s comments, and the revised sections are highlighted in yellow.

We hereby resubmit our manuscript for a secondary evaluation. Thank you again for considering our paper.

Sincerely,

Akiyo SASAKI-OTOMRU

**Journal Requirements:**

**1. Please ensure that your manuscript meets PLOS ONE's style requirements, including those for file naming. The PLOS ONE style templates can be found at**

**https://journals.plos.org/plosone/s/file?id=wjVg/PLOSOne_formatting_sample_main_body.pdf and**

**https://journals.plos.org/plosone/s/file?id=ba62/PLOSOne_formatting_sample_title_authors_affiliations.pdf**

Response

Thank you for your valuable comment. We have revised our manuscript to comply with PLoS ONE’s style requirements, including to the title page, reference format, and placement of Table 2.

**2. Thank you for stating the following financial disclosure: This work was supported by the 31st Foundation for Total Health Promotion and the Grant-in-Aid for Young Scientists (B), under Grant number. 17K17508 (KAKENHI).**

**Please state what role the funders took in the study. If the funders had no role, please state: ""The funders had no role in study design, data collection and analysis, decision to publish, or preparation of the manuscript.""**

**If this statement is not correct you must amend it as needed.**

**Please include this amended Role of Funder statement in your cover letter; we will change the online submission form on your behalf.**

Response

Thank you for your insightful comment. In our study, the funders had no role. We have stated in our cover letter: "The funders had no role in study design, data collection and analysis, decision to publish, or preparation of the manuscript."

**3. In the online submission form, you indicated that the dataset generated and/or analyzed during the current study are not publicly due to ethical considerations. The data that support the findings of this study are available from the corresponding author upon reasonable request.**

**All PLOS journals now require all data underlying the findings described in their manuscript to be freely available to other researchers, either 1. In a public repository, 2. Within the manuscript itself, or 3. Uploaded as supplementary information.**

**This policy applies to all data except where public deposition would breach compliance with the protocol approved by your research ethics board. If your data cannot be made publicly available for ethical or legal reasons (e.g., public availability would compromise patient privacy), please explain your reasons on resubmission and your exemption request will be escalated for approval.**

Response

Thank you for your perceptive comment. This study has sensitive participant information and cannot be released publicly due to ethical and legal restrictions imposed by the Ethical Committee of Yokohama City University School of Medicine. All data supporting the findings of this study are available upon reasonable request to the Department of Medical Education (inamorim@yokohama-cu.ac.jp), Yokohama City University School of Medicine. This information is also described in the online submission form.

**4. Please review your reference list to ensure that it is complete and correct. If you have cited papers that have been retracted, please include the rationale for doing so in the manuscript text, or remove these references and replace them with relevant current references. Any changes to the reference list should be mentioned in the rebuttal letter that accompanies your revised manuscript. If you need to cite a retracted article, indicate the article’s retracted status in the References list and also include a citation and full reference for the retraction notice.**

Response

Thank you for your important comment. We have replaced the reference to Jakicic et al. (2016) with Zhou et al. (2020) for reference number 12. Additionally, we have corrected the DOI for reference number 24, which is now listed as number 25 in the revised manuscript.

We have revised males and females to men and women in Table 1. Furthermore, we have used “deep-sleep duration” and “body-movement duration” with hyphen to maintain consistency.

**Reviewers' comments:**

**Reviewer #1:**

**Please provide a scale that describes the relative strength of correlation.**

Response

Thank you for your meaningful comment. We have added the relative strength of correlation in the Statistical analysis section (lines 137-139). Following the addition of reference number 22 in the revised manuscript, the subsequent reference numbers have been updated accordingly.

**Some items in the table are not aligned correctly which may simply be a function of the size of the table.**

Response

Thank you for pointing this out. We have corrected the row formatting in Table 1.

**As noted earlier you have some statistically significant correlations that may not be that clinically meaningful.**

Response

Thank you for your thoughtful comment. We have added the interpretation of correlations in the Results section (lines 155-163).

**“Strongest” but still may not be all that meaningful clinically.**

Response

Thank you for bringing this to our attention. We have revised the relevant part in the Results section (lines 177-179).

**Prior to restarting the purpose of the study please also restate the main problem you are attempting to address and the need for this study**

Response

We appreciate your attention to this matter. We have added the main problem and our hypothesis at the beginning of the Discussion section (lines 186-187)

**This statement “decreased in correlation” is too general/broad/vague.**

Response

Your meaningful comment is duly noted. We have revised the text as suggested (lines 187-188).

**A major methodological limitation is that correlation and regression only looks at relationships and not cause/effect**

Response

Your insightful comment is recognized. We have revised the limitations paragraph in the Discussion section in response to your comment (lines 233-241).

**Reviewer #2: The title "Association of home blood pressure with sleep and physical and mental activity, assessed via a wristwatch-type pulsimeter with accelerometer in adults" is not compatible whith the content of this manuscript.**

Response

Thank you for highlighting the title. Our previous research focused on blood pressure; however, the present manuscript examines the relationship between sleep and activity data, assessed using a wristwatch, and health status. Therefore, we have titled it: “Relationship of sleep and activity, assessed via a wristwatch-type pulsimeter with accelerometer, with health status in community-dwelling older adults: A preliminary study.”

**How the sample size was calculated?**

Response

Your insightful comment is appreciated. We have added an explanation of the power analysis for sample size calculation in the Discussion section (lines 235-238).

**What is the main research problem and what are the hypotheses? Dependent and independent variables need to be established.**

Response

We appreciate your valuable question. To examine the nature of sleep-activity patterns in the older population, we utilized PS as the dependent variable. Independent variables were selected based on an extensive body of literature exploring factors influencing sleep and activity. We have added our hypothesis in the Introduction section (lines 69-71).

**Why the authors did not collect information on the nutritional status, which could notably influence frailty.**

Response

We agree with your suggestion and have already included this information in the limitations section (lines 242-243).

At the beginning of the study, we assessed nutritional status using the BDHQ (Brief-Type Self-Administered Diet History Questionnaire). However, because of the substantial burden of the BDHQ’s 80-item questionnaire on participants, and given that initial results indicated good nutritional status, we decided to discontinue its use.

**In male participants, physical activity duration correlated positively with bodily pain. Could the authors explain that?**

Response

We appreciate your meaningful question. A higher bodily pain score indicates that activity is not interrupted by pain; therefore, we believe our results are consistent.

**Multilinear regression analyses were conducted to explore the associations between demographic factors and body-movement duration. Why the autors chose so kind of variables for this analises?**

Response

You have raised an important question. Because of the limited number of subjects, we selected five to six variables. Based on previous research, we chose variables expected to be associated with activity duration. Therefore, we selected BMI, hypertension, PSQI, and KCL as predictor variables, with age and gender as covariates. These points have been added (lines 176-177).

**The results showed that body movement duration decreased in correlation with the higher risk of frailty in older adults after adjusting for sex. I dont understand this conclusion and it is not supraising probably.**

**The conclusions are over-interpretation.**

Response

Thank you for pointing this out. By clearly stating our hypothesis and revising the limitations, we believe our manuscript is now more understandable. In addition, we have revised the Conclusions section to avoid over-interpretation (lines 248-249). We hope you understand our rationale for these decisions.
